# MRXCAT2.0: Synthesis of realistic numerical phantoms by combining left-ventricular shape learning, biophysical simulations and tissue texture generation

**DOI:** 10.1186/s12968-023-00934-z

**Published:** 2023-04-20

**Authors:** Stefano Buoso, Thomas Joyce, Nico Schulthess, Sebastian Kozerke

**Affiliations:** Institute for Biomedical Engineering, ETH Zurich and University Zurich, Zurich, Switzerland

**Keywords:** Cardiac magnetic resonance, Biomechanical modeling, Image synthesis, Population variability, Strain

## Abstract

**Background:**

Standardised performance assessment of image acquisition, reconstruction and processing methods is limited by the absence of images paired with ground truth reference values. To this end, we propose MRXCAT2.0 to generate synthetic data, covering healthy and pathological function, using a biophysical model. We exemplify the approach by generating cardiovascular magnetic resonance (CMR) images of healthy, infarcted, dilated and hypertrophic left-ventricular (LV) function.

**Method:**

In MRXCAT2.0, the XCAT torso phantom is coupled with a statistical shape model, describing population (patho)physiological variability, and a biophysical model, providing known and detailed functional ground truth of LV morphology and function. CMR balanced steady-state free precession images are generated using MRXCAT2.0 while realistic image appearance is ensured by assigning texturized tissue properties to the phantom labels.

**Finding:**

Paired CMR image and ground truth data of LV function were generated with a range of LV masses (85–140 g), ejection fractions (34–51%) and peak radial and circumferential strains (0.45 to 0.95 and − 0.18 to − 0.13, respectively). These ranges cover healthy and pathological cases, including infarction, dilated and hypertrophic cardiomyopathy. The generation of the anatomy takes a few seconds and it improves on current state-of-the-art models where the pathological representation is not explicitly addressed. For the full simulation framework, the biophysical models require approximately two hours, while image generation requires a few minutes per slice.

**Conclusion:**

MRXCAT2.0 offers synthesis of realistic images embedding population-based anatomical and functional variability and associated ground truth parameters to facilitate a standardized assessment of CMR acquisition, reconstruction and processing methods.

**Supplementary Information:**

The online version contains supplementary material available at 10.1186/s12968-023-00934-z.

## Background

In-silico phantoms of human cardiovascular anatomy and function provide a versatile tool for the testing and validation of image acquisition, reconstruction and post-processing strategies in cardiovascular magnetic resonance (CMR) [1]. Producing synthetic images from a phantom has the benefit that the resulting images have corresponding anatomical labels and functional ground-truth data, which are useful for the evaluation of the performance of a CMR pipeline. For example, the availability of a paired image-ground truth dataset is essential for a standardized evaluation of image processing tools, such as those for automatic left-ventricular (LV) segmentation, shape and strain analysis.

Available phantoms can be classified into three categories: voxel-based, analytical and hybrid. Voxel-based phantoms consist of labeled voxelised anatomical representations obtained from patients [2, 3]. These are realistic, but do not generalize to population statistics and pathological cases [4]. Analytical phantoms are based on a mathematical description of tissue structures [5]. While they are less realistic, they are more flexible in terms of definition of anatomical variations. Hybrid phantoms have been proposed to overcome the limitations of the previous two categories [6]. Although hybrid and analytical phantoms allow for morphological variation, anatomical and functional variability is mostly limited to healthy cases and function. Veress et al. [7] proposed to couple a hybrid phantom to a biophysical model of the LV to simulate both healthy and infarct conditions. However, as stated by the authors, the fitting process is time consuming and it cannot account for other pathological scenarios, such as cardiomyopathy. More recently, Segars et al. [8] proposed a methodology to couple a full heart functional model to the XCAT phantom. While this allows to simulate realistic cardiac function, it is specific to XCAT and it cannot be rapidly deployed to general pathological cases.

In the last years, solutions based on shape models (SM) (with voxelised or mesh representations) have been proposed to address the need for expressive anatomical descriptions [9–13]. While these works have shown the capability of representing dominant LV anatomical features, they did not focus on the definition of a sampling strategy to generate synthetic anatomies to capture population variability, including both healthy and pathological conditions.

Given an in-silico phantom, two main methodologies for generating CMR images can be identified. In the first approach, the signal is generated using numerical solutions of the physical equations (Bloch equations). This has been applied for cardiac and brain image synthesis [14–18]. In [1, 19] the use of signal models for specific sequences of interest has been proposed to compute the resulting image data. In [20] a dataset for a virtual population with varying acquisition parameters was generated using MRXCAT [1] and used to pre-train a segmentation network, which was subsequently fine-tuned on real images. This approach greatly reduces the amount of in-vivo images required. However, the segmentation performance degraded when there was no fine-tuning on real data as the simulated images were not completely realistic. In [16, 21, 22] it has been shown that synthetic images can be used to augment, and eventually replace, in-vivo datasets for training of neural networks, making realistic image synthesis an important tool for CMR development.

Alternative generative approaches consist of using neural networks for conditional synthesis or style transfer [23–30]. They have been used for several imaging modalities such as ultrasound [31], computed tomography [26] and magnetic resonance imaging [23–25, 32]. The reader is referred to [33] for a recent overview of medical image synthesis.

In [26, 34] a factorised representation of images has been proposed, composed of a spatial representation of the anatomy combined with a *modality* description. The latter describes how tissue structures are rendered in the image. However, the network cannot be used to generate new anatomies and it requires labelled images for training, which are costly to obtain. In [23] unlabelled CMR images were used to learn a multi-tissue anatomical model which was fit to variable anatomies by a learned deformation model. The anatomical model was then used to condition a SPADE-GAN [35] to synthesise an image volume. While this approach solved both issues of the two previous factorised representation learning approaches [26, 34], the anatomical model learnt using the network does not represent conventional tissue classes and is thus not suited as anatomical ground truth. In [24], the XCAT phantom was used as anatomical ground truth semantic labels and MR images were synthesized using a SPADE-GAN. In [36], DatasetGAN, leveraging the generator features of StyleGAN [37], was proposed to produce a large synthetic dataset of images and to also predict pixel-wise class labels. The evaluation of this method has demonstrated that a segmentation network trained with datasets from DatasetGAN outperforms previous semi-supervised methods and is on par with the same network trained fully-supervised on a real dataset. Similarly, SemanticGAN [38] was developed to simultaneously generate both synthetic images and corresponding segmentation labels using StyleGAN2.

While physics-based approaches allow for better control over the parameters related to image generation with respect to style transfer approaches, they produce less realistic appearance. In [39] intra-organ texture for bones and organs was proposed to improve the realism of images generated with signal models. This approach, however, has not yet been applied to CMR image synthesis.

The present work proposes MRXCAT2.0 to address the two main limitations of in-silico phantoms: reduced variability and lack of realism. Realistic LV anatomy and function are generated by coupling a statistical shape description with a biophysical model. Surrounding tissue structures are generated with the XCAT model. Tissue maps of proton density (PD), longitudinal and transverse relaxation times (T1, T2) are assigned to image labels using a neural network trained to maximize the similarity of the background with the target appearance of real CMR images. Synthetic images are then generated using MRXCAT2.0 and used to assess the performance of published CMR processing methods [40, 41] against known ground truth of healthy and pathological cardiac function as a use case.

## Methods

**Fig. 1:**
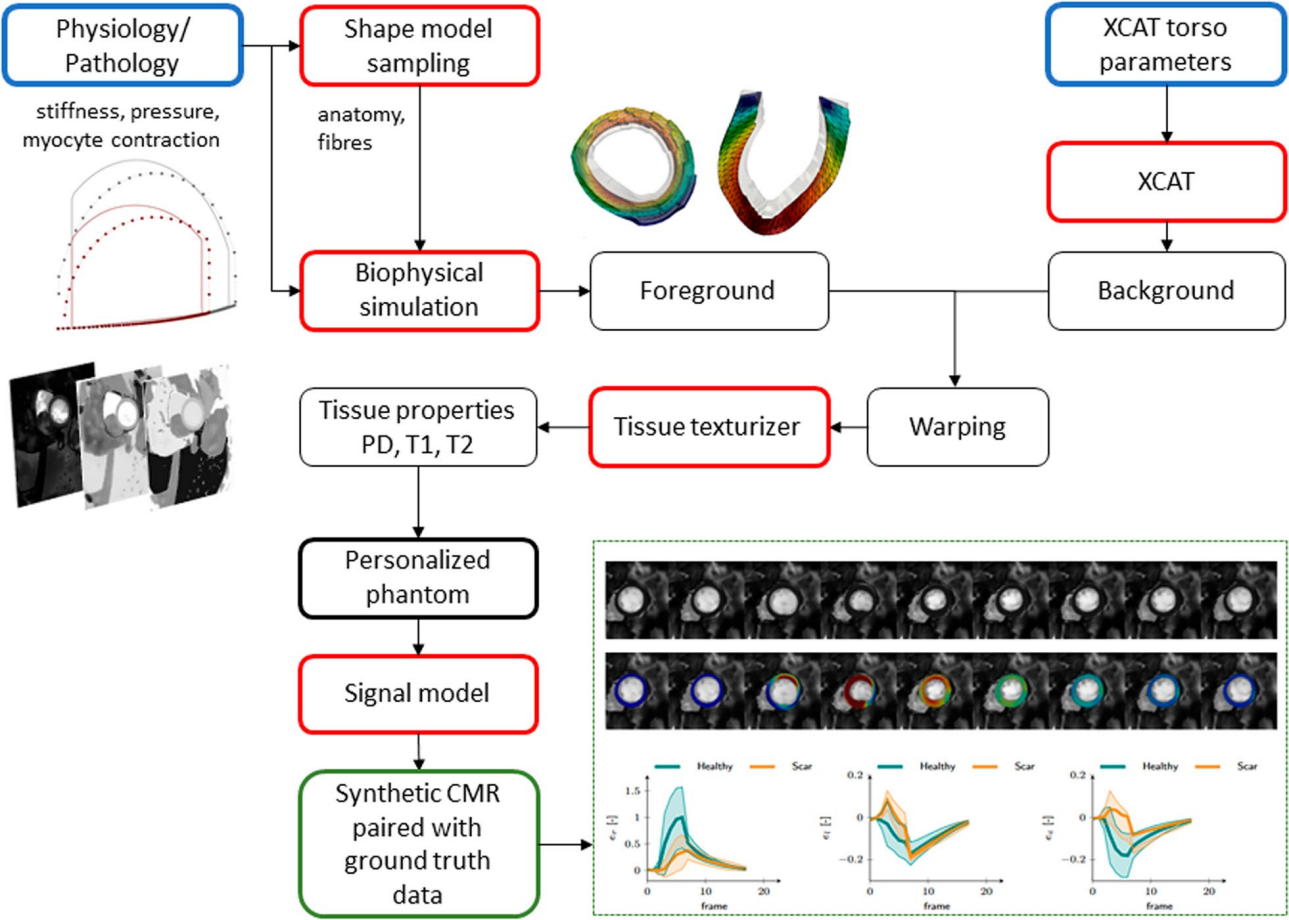
Schematic visualization of MRXCAT2.0. Input blocks are shown in blue, while the output is represented by the green box. Red boxes represent the pieces of the method (and corresponding software implementation) that are connected via the input/outputs to/from the black boxes

The full method of MRXCAT2.0 is schematically shown in Fig. 1. In the figure, red boxes correspond to the parts of the methods that are connected to each other via input/outputs of the black boxes. The final outputs are the synthetic CMR images paired with ground truth data (green box). The starting points are the two inputs: the selection of the (patho)physiological characteristics of anatomy and function and the parameters for the XCAT phantom (blue boxes). The (patho)physiological status is used to define the corresponding anatomy and tissue micro-structure from the statistical shape model and the appropriate physiological parameters (tissue stiffness, pressure loading, myocyte contraction) for the biophysical simulation that generates the image *foreground*, i.e. the LV shape and its change over the cardiac cycle. The XCAT parameters are used to define the torso anatomy and the displacement field describing the contraction of the other three cardiac chambers. This is referred to as the *background* of the image. The background tissue masks are warped to match the foreground and the resulting tissue maps are the input to the texturizer for the calculation of tissue properties (PD, T1, T2) and the definition of the final phantom. These properties are used as input to the signal model to generate synthetic CMR images associated with the input parameters and compliant with fundamental LV biomechanics.

### Left-ventricular population shape model

The LV SM was defined using the anatomies from the Multi-Modal Whole Heart Segmentation (MMWHS) dataset [42–44] as built in our recent work [13].

**Fig. 2:**
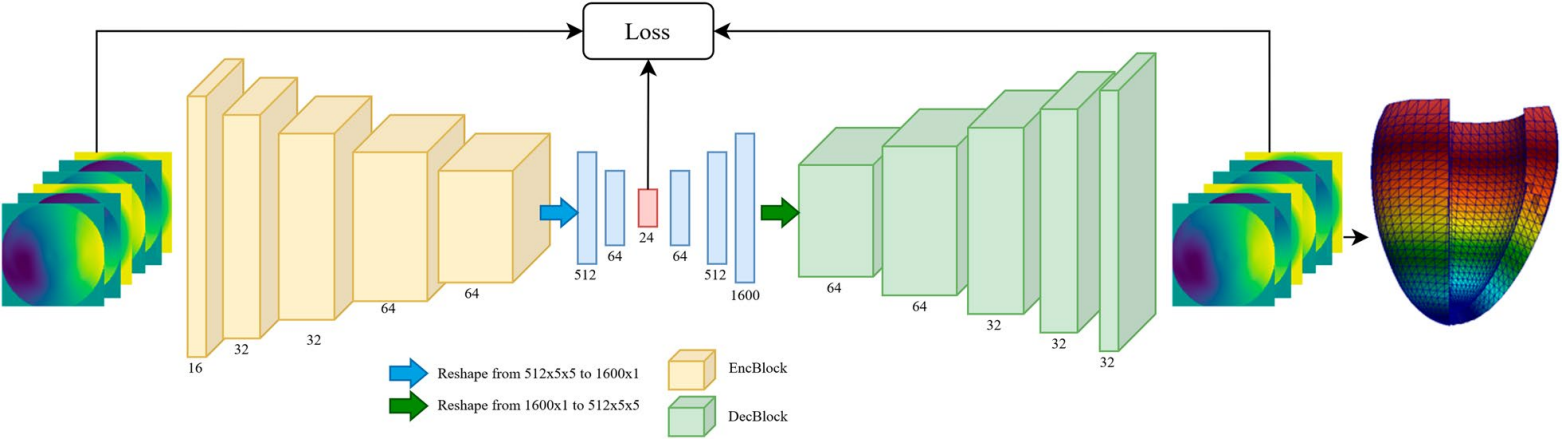
Convolutional variational autoencoder structure. The input is a 6-channel 128 × 128 pixel image representing the three-dimensional coordinates of epicardium and endocardium. Each encoder block (EncBlock) features a 2D convolutional layer (kernel size of 3, stride 1), batch normalization and a ReLu activation function. Each decoder block  (DecBlock features an upsampling bilinear layer of factor 2, a 2D convolutional layer (kernel size of 3, stride 1) and a ReLu activation function, which is not used in the last block. A ReLu activation function is also used for the linear layers. The red box represents the latent space variables

A convolutional variational autoencoder (VAE) [45] was used to identify a low-rank representation of epicardium and endocardium coordinates (see Additional file 1 for details). The network structure is shown in Fig. 2. Each variable of the low-rank representation is associated with a normal Gaussian probability distribution, which is sampled to generate synthetic realistic endocardial and epicardial shapes from which it is possible to generate a volumetric mesh [13].

The expressiveness of the SM was assessed using an additional dataset, the Automated Cardiac Diagnosis Challenge (ACDC) [46]. End-systolic images were meshed using our recently published method [12] and the accuracy of the reconstruction with the SM was evaluated as the average distance between corresponding endocardial and epicardial points in the original and reconstructed meshes. Additionally, a k-means clustering algorithm [47] was used on the latent space representation of the ACDC meshes with three target clusters to identify sampling regions of the latent space for the (patho)physiological conditions labelled in the ACDC dataset (healthy (NOR), dilated (DCM), hypertrophic (HCM)). Classification accuracy of healthy, DCM and HCM was evaluated against the clinical labels. The centres of the clusters were then used as reference anatomies for showcasing the method proposed in this work.

### Cardiac functional model

The biophysical model for the LV is based on our previous work on cardiac mechanics [13] and material modelling [48–51]. A technical description is presented in the Supplementary material and in [13].

The response of the LV to the systemic pressure loading depends on the contribution of a passive and an active component. The passive component was described by the Holzapfel-Ogden model [52] defined as a function of tissue shear moduli and fiber orientations. In our approach, the evolution of the active contribution was simulated as in [53–55]. In the model, the pericardial sac was simulated by allowing for longitudinal motion of the points but constraining epicardial radial displacement. Endocardial pressure was simulated by coupling the ventricular model to a simplified lumped-parameter model of systemic circulation [13].

### Image foreground generation

The functional model was personalized to physiological and pathological conditions of interest for the generation of the image foreground, i.e. to simulate ground-truth cardiac function. In a first step, a synthetic anatomy was sampled from the corresponding cluster (e.g. NOR, DCM, HCM). Then, material properties, fiber orientations and maximum active stress were selected to describe the target cardiac function. The LV micro-structure was defined using linear transmural laws as in [13].

Reference healthy values for the passive tissue response and potential propagation velocities were taken from [56], while values for DCM and HCM cases were obtained by defining the material coefficients between 5 and 10 times larger than in the normal case [57].

Anatomical details can be further modified by adding localized anatomical defects to any of the geometries thanks to the physiological parametrization associated with the shapes. The corresponding variations of wall thickness, mechanical and electrophysiological parameters were automatically adjusted, gradually transitioning from healthy to diseased tissue (see Additional file 1). In this work an elliptical scar at the free wall was considered, but any approach could be adopted here.

### Image background generation

The shape and functional models described in the previous sections were used to generate time-resolved 3D LV meshes that were voxelised and sliced to produce the corresponding LV tissue masks. These were then augmented by including tissue labels for the right ventricle (RV), atria and other organs using the XCAT software [6].

Each two-dimensional (2D) slice generated with the XCAT phantom was warped such that the LV epicardial contours from XCAT matched those of the epicardium from the masks generated by  LV deformations. The surrounding tissue was deformed accordingly by smooth interpolation. The approach can also account for breathing motion from XCAT. Details are presented in the Additional file 1. This coupling approach does not require modifications to the XCAT code (essentially, a self-contained additional step is added between anatomy and image generation) and, hence, keeps all functionalities of the software.

### Tissue properties definition

A neural network was used to assign textured tissue properties to the many-tissue maps combining foreground and background. A dataset of paired many-class segmentation masks and corresponding tissue-property images (i.e. images where PD, T1, and T2 values are known for every pixel) is required to train such a network. To our knowledge, there is no large dataset of tissue-property images available (even ignoring the requirement of corresponding many-class segmentation masks). Such a paired dataset was therefore synthetically generated and then used for training.

**Fig. 3:**
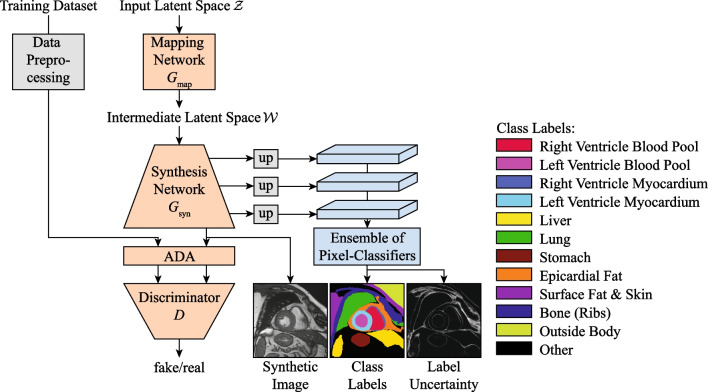
CMRGenNet structure. A StyleGAN2 with Adaptive Discriminator Augmentation (ADA) (orange blocks) generates realistic CMR images while the label generator branch (light blue blocks) predicts a semantic segmentation and also provides a segmentation uncertainty estimate as a byproduct. Note that the two branches are trained independently. In particular, there is no gradient back-propagation from the semantic label into the StyleGAN2 architecture. In addition, the mapping of class labels to colors is provided on the right

First, a CMR generative model (CMRGenNet) based on StyleGAN2 with Adaptive Discriminator Augmentation [37, 58] was trained on the ACDC dataset. Then, using the method proposed in DatasetGAN [36], the CMRGenNet was augmented with an additional branch to produce many-class labels for all generated images (see Fig. 3, technical details in the Additional file 1).

The CMRGenNet was used to generate a dataset of 8640 synthetic CMR images and corresponding 12-class segmentation masks, which were then utilized to train the MultiClassNet, a UNet [59], to predict multi-class segmentation masks from real CMR images. The MultiClassNet was then used to perform multi-class segmentation on end-systolic (ES) and end-diastolic (ED) images from the ACDC dataset.

For these segmentations, PD, T1 and T2, maximising the similarity with the corresponding image, were computed using an analytic closed-form expression of the balanced steady-state free precession (bSSFP) sequence, used to acquire the ACDC dataset (details in the Additional file 1). The same equation is used in the MRXCAT software.

This process yielded a paired dataset of 1800 parameter maps produced directly from the segmentation masks, and the corresponding detailed texture maps produced by the optimisation. As a final step, the texturizer, TextNet, was trained to map initialized uniform parameter maps to textured parameter maps. The TextNet architecture was based on a UNet.

The final images were post-processed such that any texture was removed from the LV myocardium. This is justified by the relative uniformity of image signal in the myocardium of real CMR images and the need for removing tissue property variations at the border of the myocardium resulting from partial-voluming effects. All tissue properties were then warped according to label deformations over the cardiac cycle to preserve the consistency of the anatomical details of the images.

### Synthetic CMR image generation

The resulting anatomical phantoms with corresponding texturized tissue properties were used to generate cine CMR images in MRXCAT [1]. For the use cases presented here, 2D bSSFP acquisition parameters were: repetition time TR = 3.0 ms, echo time TE = 1.5 ms, flip angle of 60°, and a signal-to-noise ratio (SNR) of 30. Eight surface coils and a Cartesian trajectory were simulated. The signal of the image was generated using the closed-form expression of the bSSFP signal equation implemented in MRXCAT, which assumes steady-state properties. As a final note, we highlight that the tissue phantom was generated at higher resolution than the target image resolution to accommodate partial voluming effects due to the limited bandwidth of the CMR encoding process.

### DeepStrain analysis

The paired ground-truth and images data generated in this work were used as input to the DeepStrain framework [40, 41]. DeepStrain leverages a network for segmentation (CarSON) and one for cardiac motion estimation (CarMEN). The networks were trained on the ACDC datasets, which were also used in our work to define TextNet. CarSON and CarMEN predictions were used as input to an additional network that computes the corresponding LV strain. To be used in these networks, the images of this work were intensity-normalized and resampled to an isotropic in-plane resolution of 1.25 mm and a total number of 16 slices. They were then cropped around the LV mask to obtain 128 × 128 × 16 pixel images.

## Results

### Left-ventricular population shape model

**Fig. 4:**
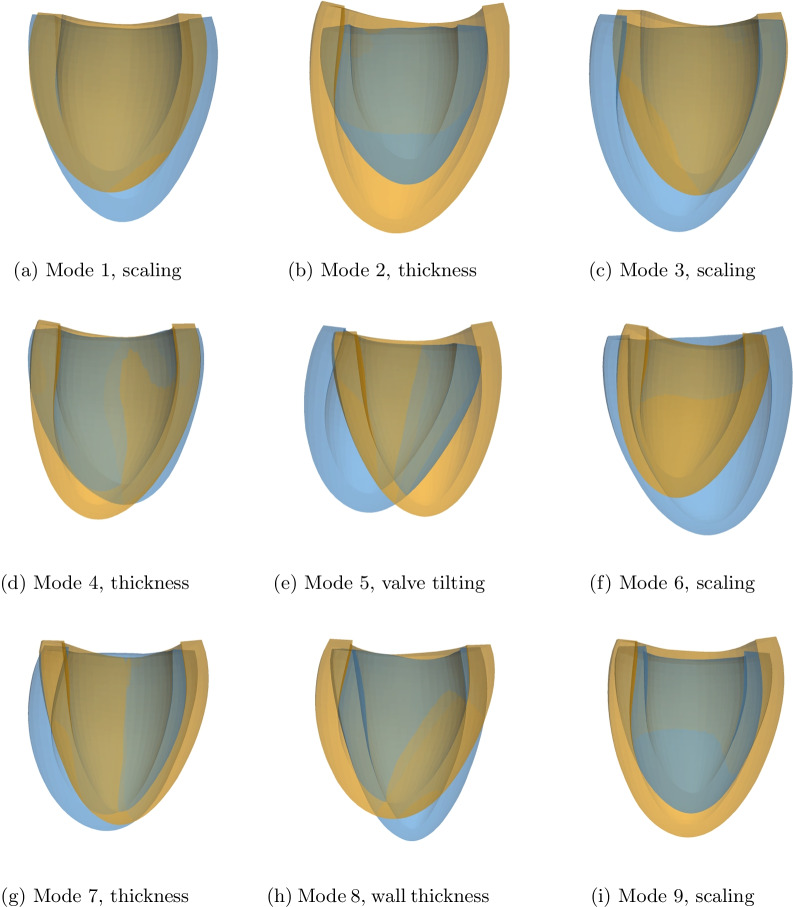
Anatomical features defined by sampling the shape model (SM) latent space. For each of the panels **a**–**i**, one variable of the latent space is sampled at -3 (orange shape) and + 3 (blue shape) standard deviations of their normal probability distribution, while all other variables are set to zero (e.g. their mean). This visualizes the anatomical variability encoded with each latent space variable. When sampling new anatomies, more than 99.7% of the shapes will lie within the bounds visualized

Figure 4 shows the SM features encoded with selected latent variables sampled at ± 3σ of the normal probability distribution defined with the VAE. These modes are associated with an identifiable physiological interpretation: global shape scaling (Fig. 4a, c, f, j), valve plane tilting (Fig. 4e), sphericity and wall thickness (Fig. 4b, d, g, h).

**Fig. 5:**
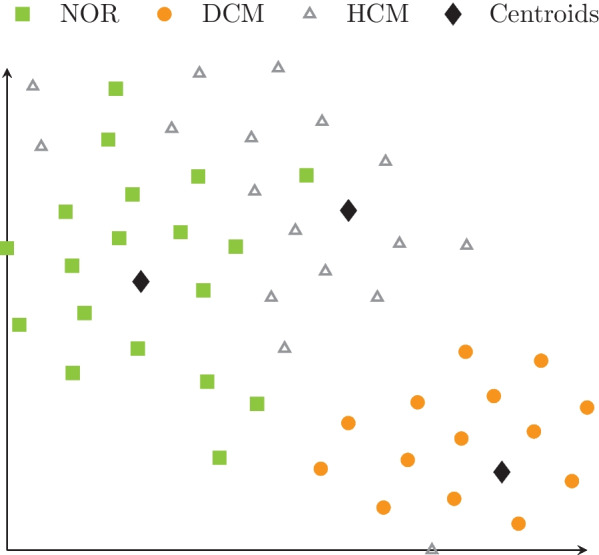
Visualization of the latent space variables of the shape model for the Automated Cardiac Diagnosis Challenge (ACDC) dataset. Anatomies are color coded according to the clinical label: healthy (NOR, green squares), hypertrophic cardiomyopathy (HCM, grey triangles) and dilated cardiomyopathy (DCM, orange circles). Black symbols show the clusters' positions calculated using the k-means algorithm on the latent-space variable vectors. Note that due to the non-linear nature of the embedding, the cluster means do not necessarily match the barycenter of the point clouds

**Fig. 6:**
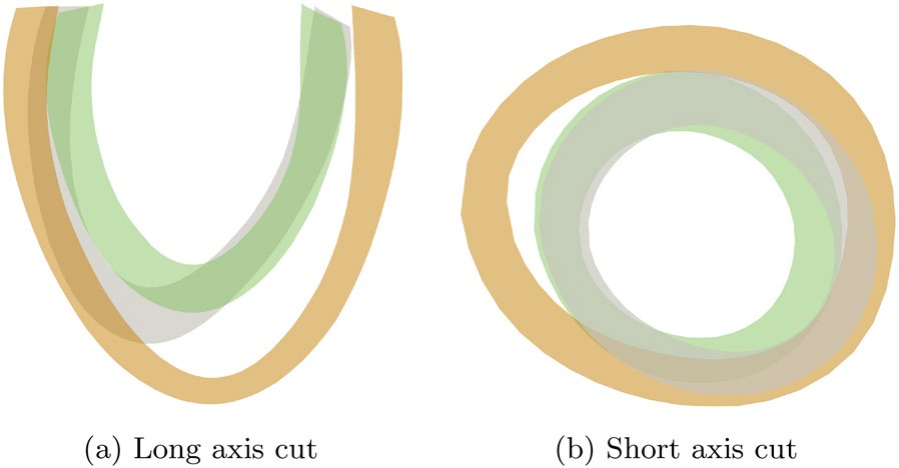
Long (**a**) and short (**b**) axis cuts of the anatomies corresponding to the healthy (green), hypertrophic cardiomyopathy (grey) and dilated cardiomyopathy (orange) cluster centers of the ACDC dataset

The average shape reconstruction error of the ACDC dataset was 6.5 ± 1.0 mm with split errors of 6.0 ± 1.0 mm, 4.5 ± 0.6 mm, and 8.0 ± 1.5 mm for healthy, DCM and HCM anatomies, respectively. A 't-SNE' map [60] was used to reduce the latent space vector dimension of the ACDC dataset to 2D and allow for the visualization of the anatomical clustering in Fig. 5. Clinical labels are shown in the figure as healthy (NOR, green squares), DCM (orange circles) and HCM, (grey triangles). The anatomies corresponding to the clusters shown in Fig. 5 (black diamonds) are visualized in Fig. 6. The accuracy of the classifier evaluated on these shapes was 0.86.

### Cardiac functional model

The simulation of LV function with the biophysical model provided a good representation of both physiological and pathological cases: LV mass were 90 g, 140 g, 100 g and 85 g for NOR, DCM, HCM and infarcted cases, respectively. ED and ES volumes were 145 ml/70 ml, 270 ml/180 ml, 156 ml/92 ml and 159 ml/80 ml for NOR, DCM, HCM and infarcted cases, respectively; resulting in LV ejection fraction values of 51%, 34%, 41% and 49%, respectively. The corresponding shape average peak systolic radial/longitudinal/circumferential strains (e_r_/e_l_/e_c_) were: 0.78/− 0.17/− 0.14 (NOR), 0.45/− 0.10/− 0.15 (DCM), 0.50/− 0.14/− 0.13 (HCM) and 0.95 (0.30)/− 0.18 (− 0.17)/− 0.18 (0.01) remote(scar) regions of the infarcted case.

### Tissue property definition

**Fig. 7:**
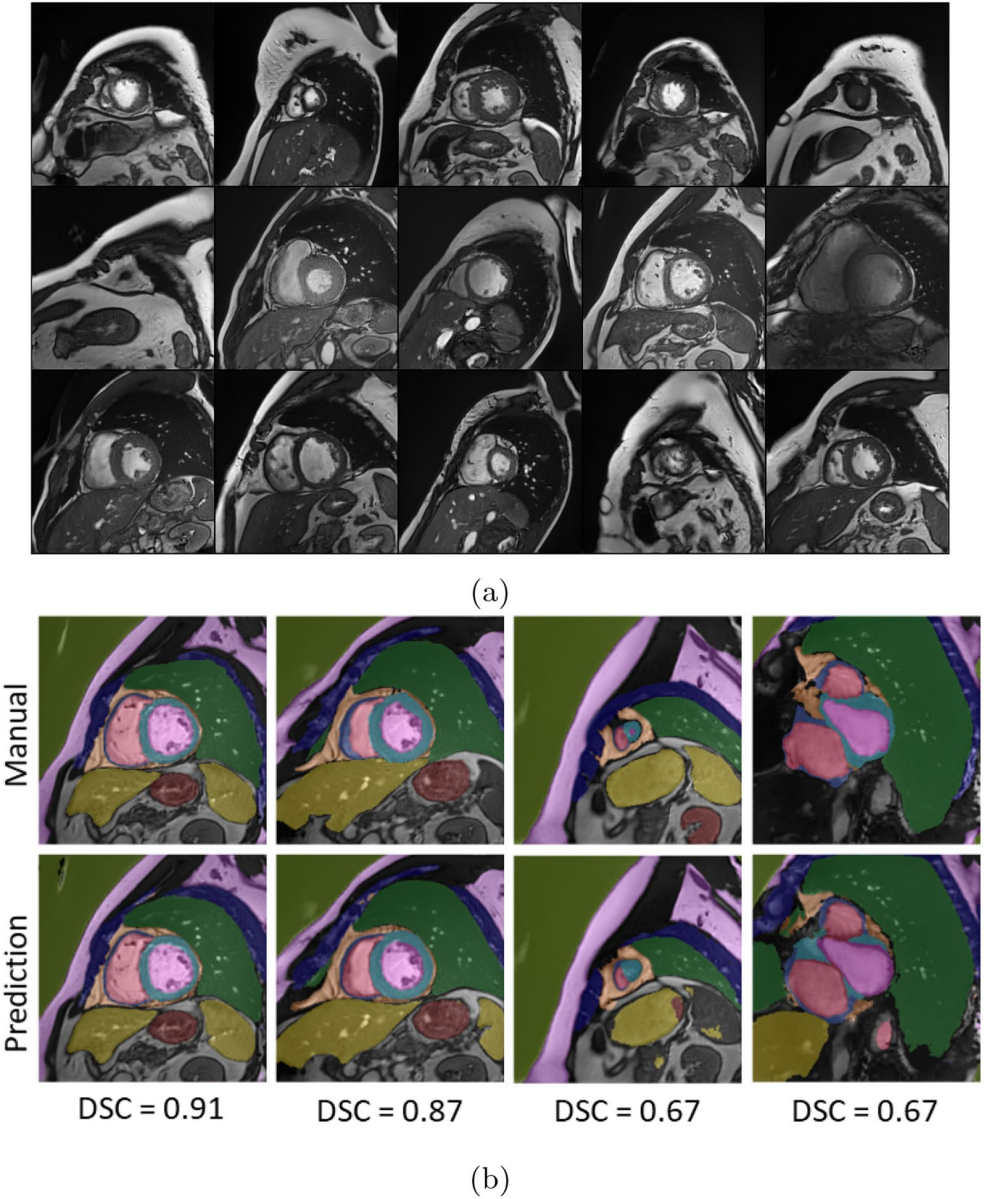
Images and multi-class labels of the CMRGenNet branches. In (**a**) 15 synthetic images are shown and in (**b**) manual annotations over 5 images (top row) are compared with predicted multi-class labels from the semantic segmentation branch (bottom row). Below each column the corresponding Dice score (DCS) between the manual and predicted annotations is reported. It is noted that these examples correspond to the best (first two columns) and worst (last two columns) predicted cases

Figure 7a shows 15 synthetic images generated with CMRGenNet and a comparison between the labels produced by CMRGenNet and our manual annotations (Fig. 7b, top and bottom rows, respectively). The CMRGenNet segmentation branch produced an average Dice score of 0.91, 0.85 for RV and LV blood pools, respectively, and 0.67 and 0.82 for RV and LV myocardium, respectively, when compared to the 10 manually annotated cases used for validation.

**Fig. 8:**
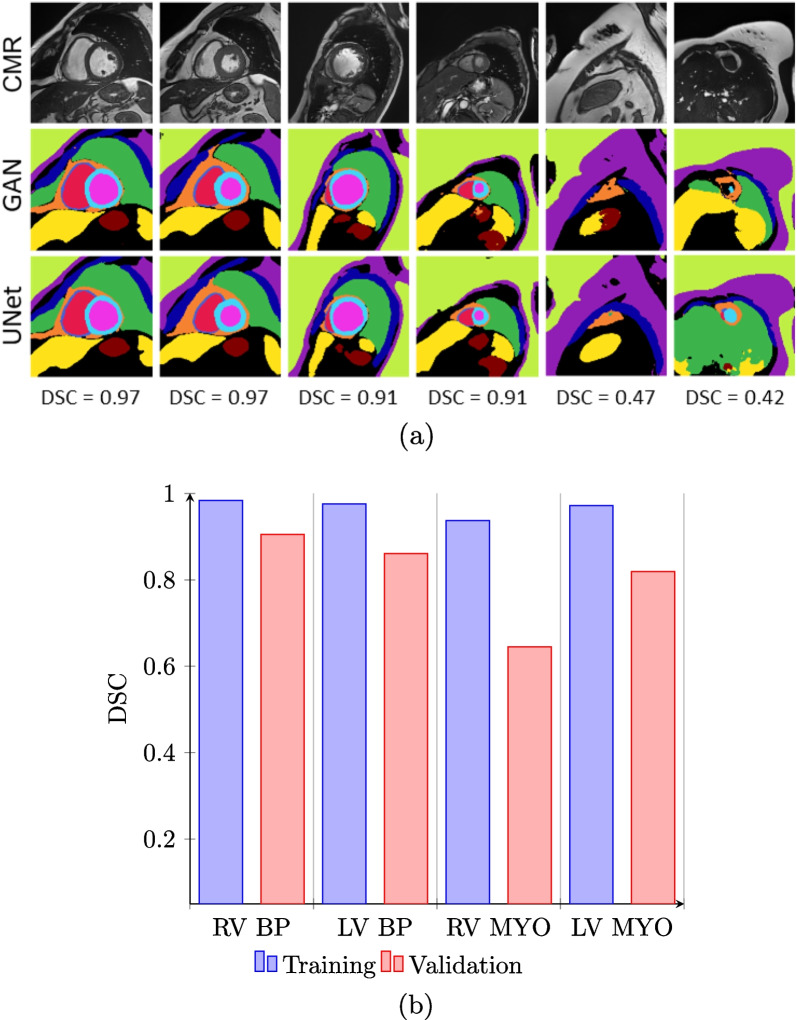
Multi-class segmentations of synthetic images. Synthetic CMR images (top row, CMR), multiclass annotation from the CMRGenNet semantic segmentation branch (mid row, GAN), and MultiClassNet predictions (bottom row, UNet) are shown (**a**). The Dice score (DCS) value for the multi-class annotations from the semantic segmentation branch is reported. It is noted that these examples correspond to the best (first two columns), average (mid columns) and worst (last two columns) predicted cases. DCS values for the segmentation branch of the MultiClassNet on labelled images used for training and validation are provided (**b**). These corresponds the synthetic images generated with CMRGenNet with the corresponding multi-class masks. Labels refer to the right ventricular (RV) and left-ventricular (LV) blood pools, RV blood pool and LV blood pool, respectively, and the RV and LV myocardium (MYO), RV MYO and LV MYO, respectively. *BP* blood pool

Figure 8 compares images and multi-class labels generated by CMRGenNet (MRI and GAN lines, respectively) with the segmentations predicted by the MultiClassNet (UNet). The MultiClassNet produced an average Dice score of 0.90, 0.86, 0.65 and 0.82 of the mask prediction for the right-ventricular (RV) blood pool, LV blood pool, RV myocardium and left-ventricular myocardium, respectively, on synthetic images generated by CMRGenNet.

### Image generation

**Fig. 9:**
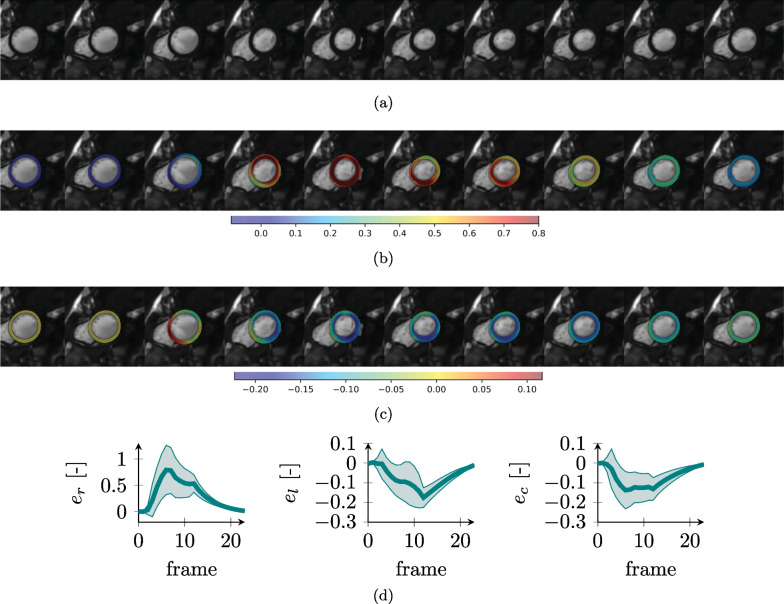
Synthetic CMR images and ground truth strains for the healthy LV. SAx image over the cardiac cycle (**a**) and overlay of radial (**b**) and circumferential (**c**) strains. Ground truth simulated strain values [mean value (solid line) and standard deviation (shaded area)] over the full anatomy for radial (e_r_), longitudinal (e_l_) and circumferential (e_c_) strains (**d**)

**Fig. 10:**
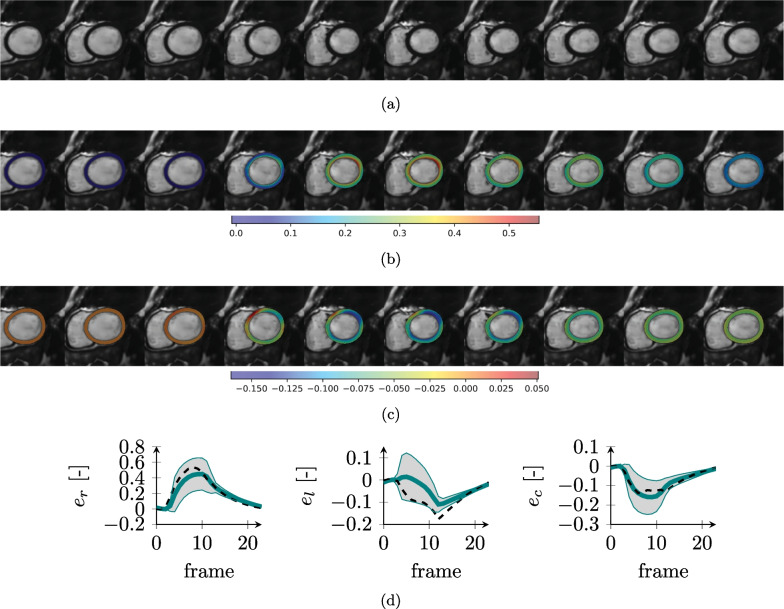
Synthetic CMR images and ground truth strains for the DCM case. SAX image over the cardiac cycle (**a**) and overlay of radial (**b**) and circumferential (**c**) strains. Ground truth simulated strain values [mean value (solid line) and standard deviation (shaded area)] over the full anatomy for radial (e_r_), longitudinal (e_l_) and circumferential (e_c_) strains (**d**). The dashed black line represents the average on the healthy anatomy for comparison

**Fig. 11:**
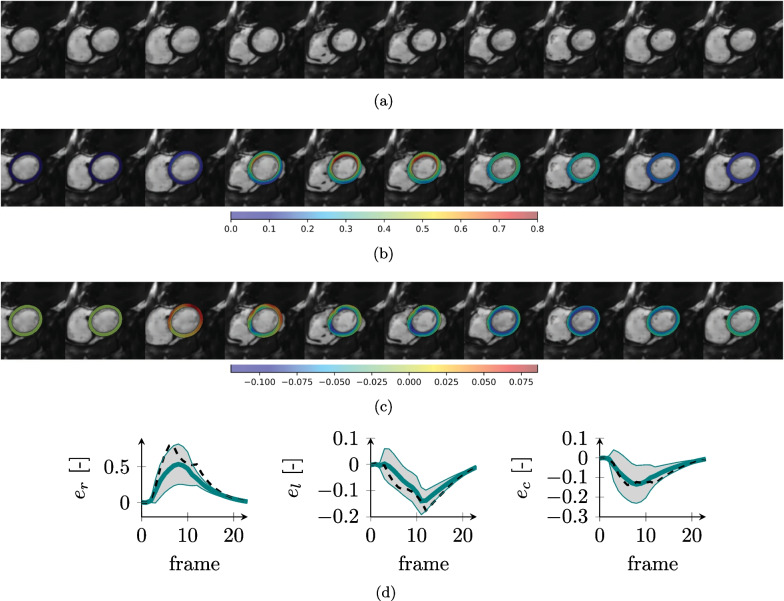
Synthetic CMR images and ground truth strains for the HCM case. SAx image over the cardiac cycle (**a**) and overlay of radial (**b**) and circumferential (**c**) strains. Ground truth simulated strain values [mean value (solid line) and standard deviation (shaded area)] over the full anatomy for radial (e_r_), longitudinal (e_l_) and circumferential (e_c_) strains (**d**). The dashed black line represents the average on the healthy anatomy for comparison

**Fig. 12:**
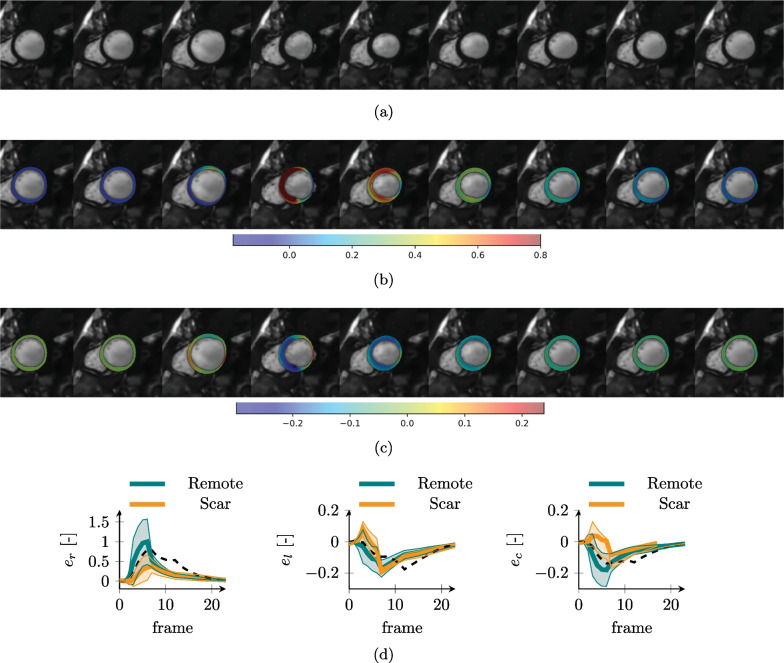
Synthetic CMR images and ground truth strains for the infarcted LV. SAx image over the cardiac cycle (**a**) and overlay of radial (**b**) and circumferential (**c**) strains. Ground truth simulated strain values [mean value (solid line) and standard deviation (shaded area)] over the full anatomy for radial (e_r_), longitudinal (e_l_) and circumferential (e_c_) strains (**d**). Strain statistics are split into scar region (orange color) and remote tissue (blue color). The dashed black line represents the average on the healthy anatomy for comparison

**Fig. 13:**
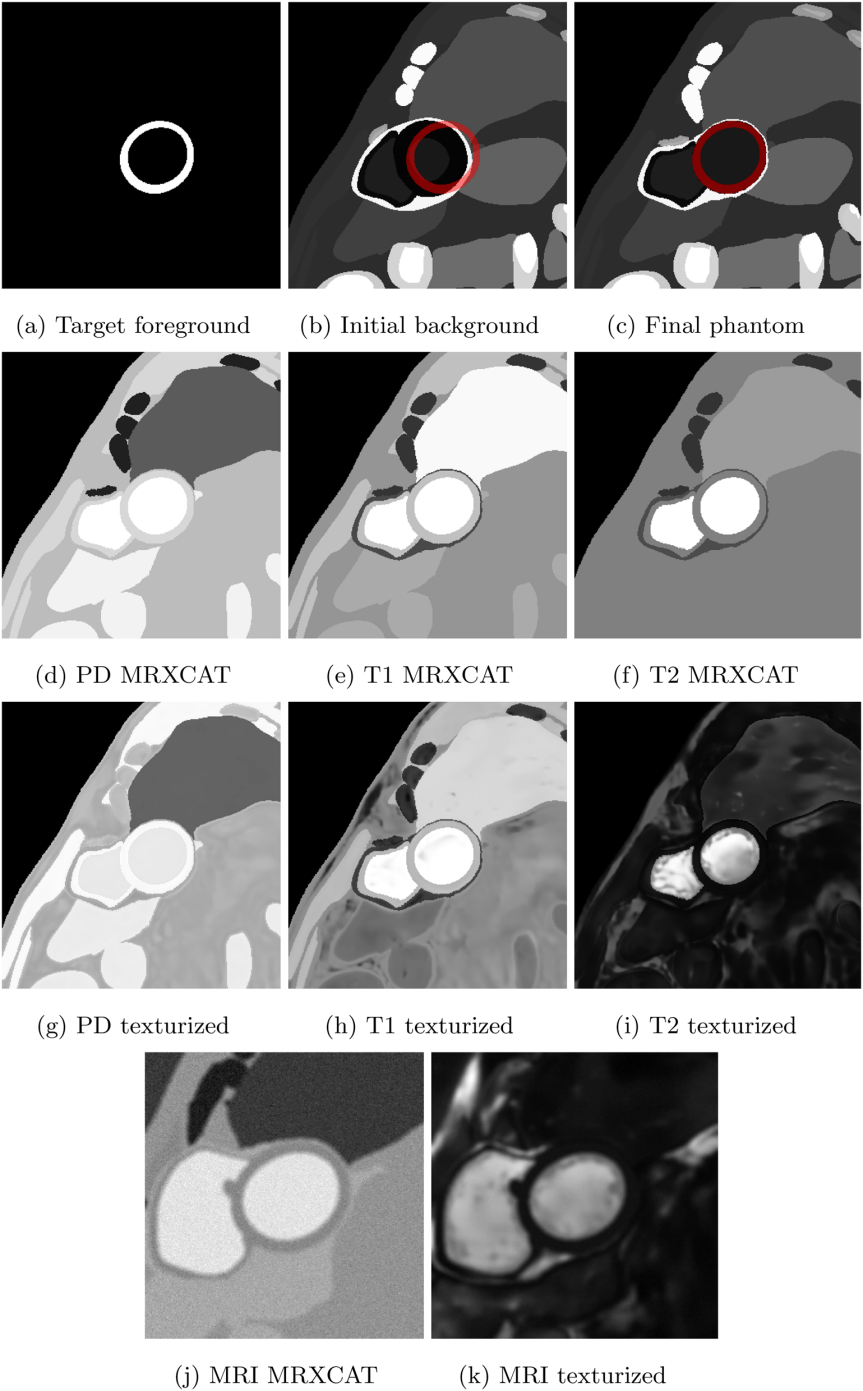
Visualization of the steps involved in the image generation process. Target LV mask from biophysical model (**a**). Initial background from XCAT (target LV mask in red) (**b**). Final phantom after background warping (target LV mask in red) (**c**), (**d**–**f**) initial tissue properties initialized from MRXCAT default values for PD, T1 and T2 respectively, (**g**–**i**) texturized PD, T1 and T2 maps after the application of TextNet, (**j**) CMR image generated with MRXCAT using the initialized tissue properties in (**d**–**f**), (**k**) CMR image generated with MRXCAT with the texturized tissue properties in **g**–**i**

Figures 9, 10, 11 and 12 show short-axis (SAx) slices of images generated with MRXCAT2.0 from the corresponding simulations overlaid with ground truth physiological strain values. Figure 13 shows the steps for the generation of the synthetic images and a comparison between the CMR images obtained without and with texturization. In the non-texturized case, the default tissue properties of MRXCAT have been considered.

### DeepStrain analysis

**Fig. 14:**
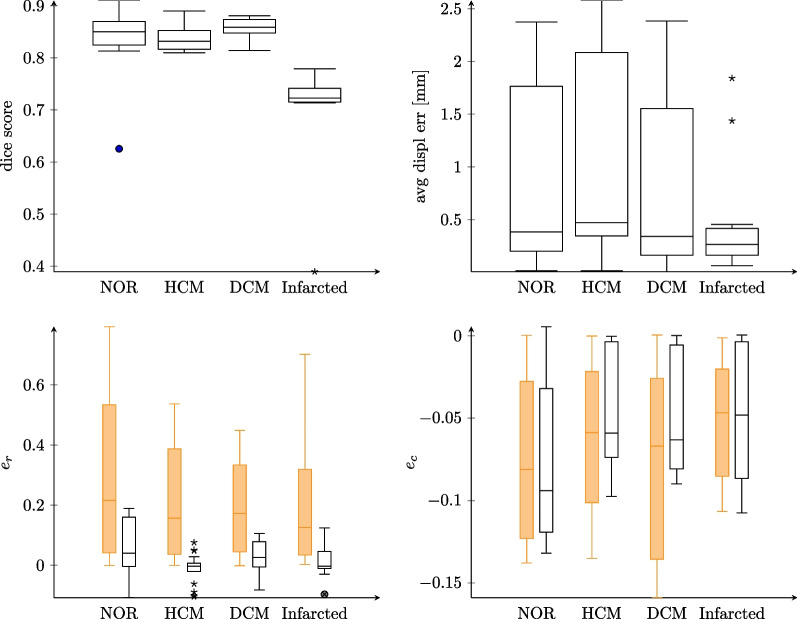
Comparison of metrics predicted by DeepStrain with ground truth synthetic values for each of the synthetic cases presented: Dice score (top left panel), displacement errors projected onto SAx plane (top right panel) and radial, e_r_, and circumferential, e_c_, strains (bottom left and right panels, respectively). All values are computed from the average of three SAx slices at the mid-ventricular position. For the strain panels, orange and black boxes refer to ground truth and DeepStrain values, respectively

Results of using the DeepStrain software are summarized in Fig. 14. On average, a Dice score of 0.82 across all cardiac phases is obtained. The lowest performance was observed for the infarcted case, in particular for the thin scar region. The average displacement error computed from the synthetic data was 1.0 ± 0.9 mm. Strain predictions showed a good agreement for circumferential strain (e_c_ average error of 0.02 ± 0.04 across all cases). Larger errors for cases with thinner walls such as DCM and infarcted cases were seen. Additionally, a general underestimation of radial strains (− 0.24 ± 0.21) was observed, with the lowest performance obtained for the infarcted case (− 0.20 ± 0.21).

The analysis of DeepStrain performance conducted by the authors in [40] showed segmentation accuracy with Dice scores of 0.89 and 0.91 for ED and ES cardiac phases, respectively, which agreed with our observed accuracy of 0.82 across all phases.

## Discussion

A methodology to extend the current XCAT torso phantoms with LV anatomical variability derived from a population-based statistical shape model has been presented. Healthy and pathological cardiac function, also including local anatomical and tissue defects, have been simulated and personalised using a biophysical cardiac electromechanical model. Tissue properties of the phantom were assigned using an image-to-image network trained to maximize the similarity of resulting images with real bSSFP CMR data. The extended phantom was then used as input to CMR image production with realistic population settings and biophysical model parameters linked to ground truth displacement and strain values. Finally, synthetic images were used to showcase their adoption in testing CMR image processing protocols.

As observed in our previous work using proper orthogonal decomposition (POD) [13], amplitude variations of a single latent variable from the VAE modulated specific anatomical features of the left ventricle. The contributions of the modes computed on the ACDC dataset showed distinct cluster values (Fig. 5) for (patho)physiological conditions as healthy, DCM and HCM. Note that, as the representation was learned in an unsupervised way, the ability to discriminate classes results directly from the disentangled nature of the learned representation. The corresponding cluster centers (black diamonds in Fig. 5) determined the anatomical shapes shown in Fig. 6. The DCM cluster center featured an ES configuration with enlarged blood pool volume (180 ml) and thin muscle walls, while the HCM case showed an increased wall size and a blood pool volume similar to the healthy case (92 ml and 70 ml, respectively).

The SM showed reasonable accuracy in the reconstruction of a new dataset, with an average error between points of 6 mm. While the error was higher in comparison to our previous POD approach [13], the VAE method determined a higher clustering accuracy (0.86) than the linear POD approach (0.78). Additionally, the normal probability distribution associated with the latent space defined by the VAE allowed for sampling of realistic synthetic geometries spanning the population variability (from the MMWHS dataset) that could be used as input for biophysical models. The use of a shape model improved the implementation proposed by [7] in two ways: it allowed for fast (a few seconds) generation of anatomies and it included the possibility of representing pathological cases. While our approach works on a discrete image and not on the parametrized anatomy as the approach proposed by [8], it is more versatile and can be coupled with any available numerical phantom.

The selection of appropriate physiological model parameters for each of the simulations allowed to obtain realistic LV cardiac functions (Figs. 9, 10, 11, 12) and ejection fractions (EF) consistent with clinical findings. The simulated healthy cardiac function (Fig. 9) had an EF of 51% and strains within the physiological range [61]. The DCM simulation (Fig. 10) resulted in reduced strains as compared to the healthy heart and an overall reduced deformation over the cycle and, consequently, a lower EF of 34%. Also, the HCM simulation resulted in a reduced EF (41%) and lower radial strains as compared to the healthy case (Fig. 11).

The infarcted LV simulation showed a preserved EF of 49%, with high strains of the remote tissue to compensate for the reduced mobility of the scar region (Fig. 12). Circumferential and longitudinal strains of the scar region also showed the typical bulging out of the muscle at the initial phases of systole, which was related to the reduced wall thickness and the rapid pressure increase in the ventricle cavity.

Images and multi-class maps produced by CMRGenNet (Fig. 7) demonstrated reasonable realism and accuracy in the predicted multi-tissue masks. Segmentation labels from the MultiClassNet, which were used as input for training the texturizer, were in good agreement with those generated from the segmentation branch of the CMRGenNet. The comparison of the CMR images in Fig. 13 demonstrated a substantial increase in realism in image appearance when using texturized tissue properties with respect to uniform fields for each mask. Also, contrarily to the previous approaches based on style transfer or image warping methods [62], our approach augmented phantom masks with tissue properties that can be used for the generation of CMR images with arbitrary sequences and parameters, allowing for higher versatility. In fact, while style transfer methods can generate very realistic images, there is no control of CMR sequence parameters, SNR, image resolution and artifacts.

In our investigation, the accuracy of the DeepStrain method in tracking LV wall displacements was 1.0 ± 0.9 mm. These errors were slightly lower than those reported in the original study (2.89 ± 1.52 mm and 1.8 ± 0.2 mm for in-vivo and synthetic images, respectively [41]). The higher accuracy for our synthetic images was mostly related to the high SNR values used in our simulations, and to the small dataset considered. In terms of segmentation performance, the lowest Dice score was observed for infarcted areas, where a thin myocardial wall was prescribed. Dice scores values very close to those reported in the DeepStrain publication for normal, HCM and DCM conditions.

Finally, a good agreement for circumferential strain predictions was found, but a significant underestimation of the radial component was observed. However, peak ES radial strains inferred in our analysis were in the range of those reported in [41] (Fig. 7a in the reference) for healthy conditions (in the range of 25%). Since for healthy conditions with EFs around 50%, radial strains between 40 and 60% are expected, we argue that radial strain underestimation could be a limitation in the DeepStrain approach. Accordingly, we believe that our synthetic images are valid.

## Limitations

The shape model, representing anatomical variability, was trained on a limited dataset. Although we observed that the resulting low-dimensional VAE representation was able to capture variations in a dataset different from the training one, we believe that higher reconstruction and training accuracy could be obtained by training the VAE on a larger clinical dataset. Additionally, while a simple scar model has been implemented, the approach could be extended to arbitrary shapes and property variations.

The biophysical model also simplified some aspects of cardiac function: the electro-physiological model was a reasonable approximation in absence of electrical pathology, but should be extended to more complex representations to account, for example, for the effect of fibrillation. Also, the circulation model did not account for the pulmonary path of the closed-loop response of the circulatory system. We also defined the LV microstructure using linear approaches, but 3D personalised representations could easily be implemented in the model [63].

The biophysical simulation was time-consuming and was responsible for more than 80% of the total computational cost. While in this work we have considered a conventional biophysical model, new approaches as the one we propose in [13] could be used to speed up the computations and reduce the computational time to a few minutes.

Given the paired dataset of simple and detailed texture maps produced using the proposed multi-step pipeline of CMRGenNet, MultiClassNet, and per-pixel tissue parameter optimisation, there is the potential to use a more powerful image-to-image model (e.g. [35]) to learn TextNet, which should allow for sharper and more realistic texturing. We also noted that the MultiClassNet did not show state-of-the-art performance in the segmentation of the LV myocardium. This aspect, however, did not represent a limitation in the method, since the multi-class masks were just used to train the texturizer and that, in the synthetic images, LV masks were defined from the biophysical model and tissue properties were manually assigned based on literature values.

We only focused on mid-ventricular 2D short-axis CMR images since the warping approach we implemented was restricted to 2D problems. Therefore, basal or apical slices could not be appropriately tracked. Further work is warranted to enable full heart simulation that could be coupled with the cardiac masks in XCAT and warped with a three-dimensional approach.

CMR images were generated from texturized tissue properties inferred from a dataset of realistic images using a simple signal model [1]. While this approach oversimplified the physical aspects related to the generation of the signal, it showed that realistic synthetic images paired with full knowledge of the biophysical ground truth could be generated. In particular, we showed that the realism gap in simulated CMR images can be significantly reduced through the use of textured phantoms. Also, we believe that results could be further improved by using a more modern GAN-based approach, such as in [64] or [35].

## Conclusions

We successfully generated paired CMR image and ground truth data of LV function using a statistical shape model coupled with a biophysical solver. Both healthy and pathological conditions, including infarcted, DCM and HCM, could be simulated. Therefore, this approach can be employed to generate representative image population datasets with associated ground truth values for the performance assessment of image acquisition, reconstruction and processing methods in CMR.

## Supplementary Information

**Additional file 1.**   Details of the technical implementation of the Methods section.

## Data Availability

The code for coupling the biomechanical simulation with the tissue phantom and the texturizer will be available upon acceptance at https://gitlab.ethz.ch/ibt-cmr-public/mrxcat-2.0 under MIT license conditions. It will include the MRXCAT2.0 code and an example case. Instructions for the download of the XCAT software will also be provided.

## Abbreviations

ACDC: Automated Cardiac Diagnosis Challenge
ADA: Adaptive Discriminator Augmentation
bSSFP: Balanced steady state free precession
CarMEN: Cardiac motion estimation
CMR: Cardiovascular magnetic resonance
CMRGenNet: CMR generative model
DCM: Dilated cardiomyopathy
ED: End diastole
EF: Ejection fraction
ES: End systole
HCM: Hypertrophic cardiomyopathy
LV: Left ventricle/left-ventricular
MMWS: Multimodal whole heart segmentation
MYO: Myocardium
NOR: Healthy
PD: Proton density
RV: Right ventricle/right-ventricular
SAx: Short axis
SM: Shape models
SNR: Signal-to-noise ratio
VAE: Variational autoencoder
