# Supplementary material for: MRXCAT2.0: Synthesis of realistic numerical phantoms by combining left-ventricular shape learning, biophysical simulations and tissue texture generation
Source: J Cardiovasc Magn Reson. 2023 Apr 20;25:25. doi: 10.1186/s12968-023-00934-z (PMC10116689; doi:10.1186/s12968-023-00934-z)
Supplement: Supplementary file 1 — Additional file 1. Details of the technical implementation of the Methods section. [file 12968_2023_934_MOESM1_ESM.pdf]

RESEARCH

MRXCAT2.0: Synthesis of realistic numerical phantoms by combining left-ventricular shape learning, biophysical simulations and tissue texture generation

Stefano Buoso[1*†], Thomas Joyce[1†], Nico Schulthess[1], Sebastian Kozerke[1]

Additional file 1

Left-ventricular shape model

The 75 cases of the Multi-Modal Whole Heart Segmentation (MMWHS) dataset [1–3] were manually corrected and mapped with a shape-adapted physiological parametrization approximating prolate ellipsoid radial, x_r, circumferential, x_c, and longitudinal, x_l, coordinates [4]. Using this parametrization, a reference mesh was registered onto each geometry, ensuring preserved mesh connectivity and anatomical correspondence. Epicardial and endocardial coordinates were then mapped onto disks with 64 pixel radius in the center of 128x128 pixel images. In total, 6 channels were obtained by the three-dimensional coordinates of the two surfaces (Fig. 2). A variational autoencoder (VAE) [5] was then trained to reconstruct the surfaces of interest and, at the same time, identify a suitable low-rank representation associated with a normal Gaussian probability distribution for each of the variables. During training, an Adam optimizer was used to minimize the KL divergence and Mean Squared Error of the reconstruction of the images. From the 6 channels of output from the decoder, epicardial and endocardial points were reconstructed and, finally, a volumetric mesh [6] was obtained. An alternative approach consists of applying Proper Orthogonal Decomposition as in [6, 7] to generate a set of optimal modes for the linear reconstruction of the dataset. In this case, however, it will be required to fit probability distributions from the resulting low-rank representations which was, instead, not necessary with a VAE approach.

*Correspondence: buoso@biomed.ee.ethz.ch
Institute of Biomedical Engineering, ETH Zurich and University of Zurich, Gloriastrasse, Zurich, Switzerland
Full list of author information is available at the end of the article
†Equal contributor

Cardiac Function Model

We denote as $\boldsymbol{\Omega}_0$ the LV reference (end-systolic) configuration with boundaries $\boldsymbol{\Gamma}_{endo}$, $\boldsymbol{\Gamma}_{epi}$, and $\boldsymbol{\Gamma}_{base}$ being the endocardium, epicardium and base, respectively. Let $\boldsymbol{\Omega}$ be a deformed configuration. The deformation gradient can be written as $\boldsymbol{F} = \nabla \boldsymbol{u} + \mathbb{I}$ where $\boldsymbol{u}(\boldsymbol{X}, t)$ is the displacement field from $\boldsymbol{\Omega}_0$ to $\boldsymbol{\Omega}$ and $\mathbb{I} \in \mathbb{R}^{3\times 3}$ is the identity tensor. The deformation gradient is split into volumetric and isochoric components [8] so that $\boldsymbol{F}_{iso} = \boldsymbol{F} J^{-\frac{1}{3}}$ represents the isochoric deformation and $J = \det(\boldsymbol{F})$ measures changes in volume.

The passive mechanical response of the LV is defined using the nearly-incompressible anisotropic strain energy function, $\mathscr{W}_p$ [9]:

$$\mathscr{W}_p(\boldsymbol{C},J)=\frac{a}{b}\left[e^{b(I_1-3)}-1\right]+\frac{a_f}{2b_f}\left[e^{b_f(I_{4,f}-1)^2}-1\right]+\frac{a_s}{2b_s}\left[e^{b_s(I_{4,s}-1)_+^2}-1\right]+\frac{a_{fs}}{b_{fs}}\left[e^{b_{fs}I_{8,fs}^2}-1\right]+\frac{K}{2}(J-1)^2, \tag{1}$$

where a, b, a_f, b_f, a_s, b_s, a_{fs} and b_{fs} are material coefficients, K is the bulk modulus penalising volume variations and

$$I_1=J^{-\frac{2}{3}}\mathrm{tr}\boldsymbol{C},\qquad I_{4,f}=J^{-\frac{2}{3}}\boldsymbol{f}_0\cdot\boldsymbol{C}\boldsymbol{f}_0,\qquad I_{4,s}=J^{-\frac{2}{3}}\boldsymbol{s}_0\cdot\boldsymbol{C}\boldsymbol{s}_0\qquad I_{8,fs}=J^{-\frac{2}{3}}\boldsymbol{f}_0\cdot\boldsymbol{C}\boldsymbol{s}_0, \tag{2}$$

are the invariants of $\boldsymbol{C}=\boldsymbol{F}^T\boldsymbol{F}$ and $\boldsymbol{f}_0\in\mathbb{R}^{3\times1}$ and $\boldsymbol{s}_0\in\mathbb{R}^{3\times1}$ are the local fibres and sheet directions of the heart tissue in the reference configuration $\boldsymbol{\Omega}_0$.
The passive stress tensor in the deformed configuration, $\boldsymbol{\sigma}_p$, is given by the Cauchy stress tensor as

$$\boldsymbol{\sigma}_p=J^{-1}\boldsymbol{F}^T\boldsymbol{S}\boldsymbol{F}. \tag{3}$$

where $\boldsymbol{S}=2\frac{\partial\mathscr{W}_p}{\partial\boldsymbol{C}}$ is the second Piola-Kirchhoff tensor defined in $\mathbb{R}^{3\times3}$.
The total stress in the LV is obtained as the sum of the contribution of the passive stress, $\boldsymbol{\sigma}_p$, and the active stress, $\boldsymbol{\sigma}_a$, determined by the myocyte contraction as

$$\boldsymbol{\sigma}_a=T_a\left(\boldsymbol{f}\otimes\boldsymbol{f}+\eta\left(\boldsymbol{s}\otimes\boldsymbol{s}+\boldsymbol{n}\otimes\boldsymbol{n}\right)\right), \tag{4}$$

where T_a is the stress in the fibre direction, η is a coefficient in the range $[0,1]$ that accounts for the contribution of the stress in the cross fibres directions and $\boldsymbol{f}=\boldsymbol{F}\boldsymbol{f}_0$, $\boldsymbol{s}=\boldsymbol{F}\boldsymbol{s}_0$ and $\boldsymbol{n}=\boldsymbol{F}\boldsymbol{n}_0$ are, respectively, the fiber, sheet and fiber-sheet normal directions in the deformed configuration.

The momentum balance equation, in the reference configuration, neglecting body forces and inertia effects, is defined as:

$$\begin{cases}\nabla_0\cdot\boldsymbol{P}=0 & \text{in } \boldsymbol{\Omega}_0,\\ \boldsymbol{P}\boldsymbol{N}=-Jp_{endo}\boldsymbol{N}\boldsymbol{F}^{-T}dA & \text{on } \boldsymbol{\Gamma}_{endo},\\ \boldsymbol{P}\boldsymbol{N}=J\sigma_{peri}\boldsymbol{N}\boldsymbol{F}^{-T}dA & \text{on } \boldsymbol{\Gamma}_{epi},\\ \boldsymbol{P}\boldsymbol{N}=J\sigma_{base}\boldsymbol{N}\boldsymbol{F}^{-T}dA & \text{on } \boldsymbol{\Gamma}_{base},\end{cases} \tag{5}$$

where $\boldsymbol{P}=J\left(\boldsymbol{\sigma}_p+\boldsymbol{\sigma}_a\right)\boldsymbol{F}^{-T}\in\mathbb{R}^{3\times3}$ is the first Piola-Kirchhoff stress tensor, $\boldsymbol{N}\in\mathbb{R}^{3\times1}$ is the surface normal vector in the reference configuration, p_{endo} is the pressure generated by the interaction of blood pool and endocardium, σ_{peri} and σ_{base} defines the effect of the pericardium and the traction stress at the base, respectively. In our model, the pericardium was modelled with linear normal springs of modulus K_p so that $\sigma_{peri}=K_p(\boldsymbol{x})\,\boldsymbol{u}\cdot\boldsymbol{N}$ [10]. At the base, σ_{base} represents the effect of the left atrium which was not included in our model. This boundary condition was modelled with linear springs with modulus K_b and rest-configuration at distance $\boldsymbol{u}_{ref}$ from the base so that $\sigma_{base}=K_b(\boldsymbol{x})\left(\boldsymbol{u}-\boldsymbol{u}_{ref}\right)\cdot\boldsymbol{N}$.

The value of T_a (4) is usually determined from electrophysiological models [11–13]. As in the work of [13], myocite activation time were computed by solving an Eikonal model specifying an anisotropic diffusion tensor. This was defined as $\boldsymbol{D}_{healthy}=d_f\boldsymbol{f}_0\otimes\boldsymbol{f}_0+d_s\boldsymbol{s}_0\otimes\boldsymbol{s}_0+d_n\boldsymbol{n}_0\otimes\boldsymbol{n}_0$, where d_f, d_s and d_n are the conduction coefficients along the fiber, sheet and fiber-sheet normal directions, respectively. The effect of the Purkinje fibre network was approximated by prescribing four entry points at the endocardial surface for the propagation of the activation wave. Alternatively, in our model, it is possible to specify uniform activation of the myocardium. Activation times were then used as a fixed parameter in the electromechanical model

to define the actuation of each point using a double Hill model [14, 15].

LV micro-structure was defined using linear transmural laws as in [6]. The particularization of the model to physiological and pathological conditions was obtained with the appropriate selection of the LV initial reference shape, tissue properties and systemic circulation parameters. The first step was the sampling of the anatomy from the corresponding cluster. Then, material properties, fiber orientations and maximum active stress, T_a, were selected to obtain the target cardiac function. Reference healthy values for the passive tissue response (1) were taken from [16]. Lastly, the diffusion coefficient along fiber directions was set to values in a similar range to those previously reported in the literature [16]. Specifically, d_f, was set to 0.4 while d_s and normal d_n, were set to 0.2 and 0.1, respectively. Previous works on the personalization of cardiac models to DCM and HCM cases have shown that these are characterised by stiffer material coefficients, i.e a, a_s, a_f, a_fs were between 5 and 10 times larger than in the normal case, and smaller physiological strains [17].

Local tissue defects, such as scars, were simulated by varying geometry and material properties in selected regions of the anatomy. In our work, scar regions were identified by an ellipse defined by

$$\frac{(x_l - x_{l,0})^2}{\Delta l^2} + \frac{(x_c - x_{c,0})^2}{\Delta c^2} < 1, \qquad (6)$$

where x_l and x_c are mesh points longitudinal and circumferential parametric coordinates, $x_{l,0}$ and $x_{c,0}$ are the longitudinal and circumferential parametric coordinates of the center of the ellipse, and Δl and Δc are the half lengths of the axis in the longitudinal and circumferential directions, respectively. All points in the scar were associated with a scar parameter $s_p = \frac{(x_l - x_{l,0})^2}{\Delta l^2} + \frac{(x_c - x_{c,0})^2}{\Delta c^2}$, while all external points were set with $s_p = 0.0$. This scar parameter was used to blend target thickness reduction, h_t, target material stiffening, p, and a target active stress reduction percentage, ΔT_a, with those of the healthy tissue. In particular, $h_p = (1 - s_p)h_t$ is the local values of thickness reduction within the scar, $m_s = 1 + (1 - s_p)p$ is the local material scaling, $\Delta T_{a,p} = (1 - s_p)\Delta T_a$ is the local reduction of active stress and $\boldsymbol{D}_p = \boldsymbol{D}_{healthy}(1 - s_p)$ is the local diffusion tensor for the Eikonal problem. Eq.s (5) were solved in FEniCs [18, 19] using linear tetrahedral elements. Endocardial pressure was computed by coupling the ventricular model to a simplified lumped-parameter model of systemic circulation as described in [6]. After the simulation, the end-diastolic configuration was identified and used as reference for the calculation of all simulated displacements. This step was used to adhere to the clinical convention where the starting phase is the end-diastolic one.

Important ground truth metrics in the development of imaging protocols are physiological strains. Given a deformation field $\boldsymbol{u}$ from the end-diastolic reference configuration $\boldsymbol{\Omega}_{ED}$ to a deformed state $\boldsymbol{\Omega}$, the Lagrangian strain tensor was defined as $\boldsymbol{E} = \frac{1}{2}(\boldsymbol{C} - \mathbb{I})$. The physiological strain, e_i in one of the physiological directions $\boldsymbol{v}_i$ was obtained as $e_i = \boldsymbol{v}_i^T \boldsymbol{E} \boldsymbol{v}_i$. In our work we considered strains in the radial, longitudinal and circumferential directions defined by the physiological parametrization, as e_r, e_l and e_c, respectively.

Image background generation

To generate proper background for each phase, the initial image generated from XCAT was first scaled so that the LV principal axis matched those of the mesh from the biophysical model, re-sampled to the original target resolution and translated so that the mitral valve plane of the LV coincided with the basal plane of the LV mesh. Image labels were first warped according to the displacement fields extracted from

XCAT. These included the displacements of the right ventricle and atria and, if requested, the motion field from breathing. In the latter, since the LV apex position during the beating cycle was zero without breathing displacement, the biophysical mesh was translated according to the movement of the apex position. Then, the LV masks were obtained from the biophysical model for each 2D planes of the XCAT image stack. Epicardial and endocardial contours of both masks (from LV mesh and XCAT) were fitted with a spline function and sampled at 60 equally spaced positions. From the corresponding points in the contours, the in-plane warping displacements of points of the XCAT masks were computed and interpolated on the full image constraining to zero the displacement of the boundary points of the image. Image labels were then warped accordingly. The displacement fields computed in these steps were also used to warp the tissue properties of the mask labels of the first cardiac phase. In this way we preserved the consistency of the tissue information over the cardiac cycle.

Tissue properties definition

The CMRGenNet was based on the StyleGAN2 with Adaptive Discriminator Augmentation (ADA) ([20, 21]) and trained on the ACDC dataset using an Adam optimiser [22] with learning rate of 2.5 $\times 10^{-5}$ for the mapping network and 2.5 $\times 10^{-3}$ for the synthesis network as well as the discriminator (Fig. 3). All activation functions were leaky ReLU with negative slope 0.2. The dataset comprised short-axis CMR images of 20 healthy and 80 pathological patients. The resolution varied from 0.7 mm to 1.9 mm and slice thickness was between 5 mm and 10 mm. Therefore, the images were transformed into a standardised representation before being used as training data. Each 2D slice was resampled to an isotropic in-plane resolution of 1.0 mm, centred slice-wise around the heart and rotated volume-wise such that the centroids of the two ventricular blood pools were always on the horizontal axis. Images were then cropped to the target resolution of 256 $\times$ 256 around the heart centroid. As a final step, the intensity of each 2D slice was normalised to the range [0; 255]. The 2D images were split for training and testing with a ratio of 0.8:0.2, respectively. After training, the model was used to generate 30 images (20 for training and 10 for testing) which were manually labeled with 10 anatomical classes (see Fig.3 for details on the classes). The labelled images were then used to train a second branch of the network predicting the corresponding multi-tissue segmentations for each synthesized image. The semantic segmentation branch was trained with the Adam optimizer with learning rate 10^{-3}. Training was run for 500 epochs with a batch size of 2.

The MultiClassNet was trained for 10 epochs using Adam with default parameters, learning rate of 10^{-3} and Dice loss as training objective. Synthetic images from the CMRGenNet were augmented with random rotations, image flips, shearing, distortion, zooming and brightness and contrast transformations using the Augmentor package [23]. The MultiClassNet was then used to segment end-systolic (ES) and end-diastolic (ED) images from the ACDC dataset. Initial tissue property values for PD, T1 and T2 were initialized with the default values in MRXCAT [24]. The per-pixel signal was then computed with the analytic closed-form expression of the balanced steady-state free precession (bSSFP) [24]. As the signal model is differentiable, a per-pixel minimization, using gradient descent, was performed to find the appropriate PD, T1 and T2 values that, for the bSSFP signal model, produced the target signal from the image. Initializing the multi-class segmentation masks using the default values from

MRXCAT determined a reasonable initial starting point that allowed the optimization to quickly converge to feasible values, i.e. values that were both close to the known organ tissue parameters, and also producing the intensity variation seen in the target image. Various prior knowledge could also be leveraged by suitably regularising this optimisation process. Here we regularised the optimisation by strongly penalising variations in PD from the initial values, while using a weak penalisation for variations in T1 and T2.

Finally, the TextNet was trained using the image-tissue values pairs obtained in the previous step with a 0.8:0.2 split for training, validation and testing, respectively. We used an using an Adam optimiser with learning rate of 1 $\times 10^{-3}$.

Author details

[1]Institute of Biomedical Engineering, ETH Zurich and University of Zurich, Gloriastrasse, Zurich, Switzerland.

References

1. Zhuang, X., Shen, J.: Multi-scale patch and multi-modality atlases for whole heart segmentation of MRI. Medical Image Analysis **31**, 77–87 (2016). doi:10.1016/j.media.2016.02.006
2. Zhuang, X.: Challenges and methodologies of fully automatic whole heart segmentation: A review. Journal of Healthcare Engineering **4**(3), 371–407 (2013). doi:10.1260/2040-2295.4.3.371
3. Zhuang, X., Rhode, K.S., Razavi, R.S., Hawkes, D.J., Ourselin, S.: A registration-based propagation framework for automatic whole heart segmentation of cardiac MRI. IEEE Transactions on Medical Imaging **29**(9), 1612–1625 (2010). doi:10.1109/TMI.2010.2047112
4. Toussaint, N., Stoeck, C.T., Schaeffter, T., Kozerke, S., Sermesant, M., Batchelor, P.G.: In vivo human cardiac fibre architecture estimation using shape-based diffusion tensor processing. Medical Image Analysis **17**(8), 1243–1255 (2013). doi:10.1016/j.media.2013.02.008
5. Kingma, D.P., Welling, M.: Auto-encoding variational bayes. arXiv preprint arXiv:1312.6114 (2013). doi:10.48550/arXiv.1312.611
6. Buoso, S., Joyce, T., Kozerke, S.: Personalising left-ventricular biophysical models of the heart using parametric physics-informed neural networks. Medical Image Analysis **71**, 102066 (2021). doi:10.1016/j.media.2021.102066
7. Buoso, S., Manzoni, A., Alkadhi, H., Plass, A., Quarteroni, A., Kurtcuoglu, V.: Reduced-order modeling of blood flow for noninvasive functional evaluation of coronary artery disease. Biomechanics and Modeling in Mechanobiology **18**(6), 1867–1881 (2019). doi:10.1007/s10237-019-01182-w
8. Bonet, J.: Large strain viscoelastic constitutive models. International Journal of Solids and Structures **38** (2001). doi:10.1016/S0020-7683(00)00215-8
9. Holzapfel, G.A., Ogden, R.W.: Constitutive modelling of passive myocardium: a structurally based framework for material characterization. Philosophical Transactions of the Royal Society A: Mathematical, Physical and Engineering Sciences **367**(1902), 3445–3475 (2009). doi:10.1098/rsta.2009.0091
10. Pfaller, M.R., Hörmann, J.M., Weigl, M., Nagler, A., Chabiniok, R., Bertoglio, C., Wall, W.A.: The importance of the pericardium for cardiac biomechanics: from physiology to computational modeling. Biomechanics and Modeling in Mechanobiology **18**, 503–529 (2019). doi:10.1007/s10237-018-1098-4
11. Roth, B.J., Wikswo, J.P.: A bidomain model for the extracellular potential and magnetic field of cardiac tissue. IEEE Transactions in Biomedical Engineering **33**, 467–469 (1986). doi:10.1109/TBME.1986.325804
12. Potse, M., Dube, B., Richer, J., Vinet, A., Gulrajani, R.M.: A comparison of monodomain and bidomain reaction-diffusion models for action potential propagation in the human heart. IEEE Transactions in Biomedical Engineering **43**, 2425–243 (2006). doi:10.1109/TBME.2006.880875
13. Neic, A., Campos, F.O., Prassl, A.J., Niederer, S.A., Bishop, M.J., Vigmond, E.J., Plank, G.: Efficient computation of electrograms and ECGs in human whole heart simulations using a reaction-eikonal model. Journal of Computational Physics **346**, 191–211 (2017). doi:10.1016/j.jcp.2017.06.020
14. Augustin, C.M., Gsell, M.A.F., Karabelas, E., Willemen, E., Prinzen, F., Lumens, J., Vigmond, E.J., Plank, G.: Validation of a 3D-0D closed-loop model of the heart and circulation – Modeling the experimental assessment of diastolic and systolic ventricular properties (2020). 2009.08802
15. Niederer, S.A., Plank, G., Chinchapatnam, P., Ginks, M., Lamata, P., Rhode, K.S., Rinaldi, C.A., Razavi, R., Smith, N.P.: Length-dependent tension in the failing heart and the efficacy of cardiac resynchronization therapy. Cardiovascular research **89**(2), 336–343 (2011). doi:10.1093/cvr/cvq318
16. Peirlinck, M., Sack, K.L., De Backer, P., Morais, P., Segers, P., Franz, T., De Beule, M.: Kinematic boundary conditions substantially impact in silico ventricular function. International Journal for Numerical Methods in Biomedical Engineering **35**(1), 3151 (2019). doi:10.1002/cnm.3151
17. Miller, R., Kerfoot, E., Mauger, C., Ismail, T.F., Young, A.A., Nordsletten, D.A.: An implementation of patient-specific biventricular mechanics simulations with a deep learning and computational pipeline. Frontiers in Physiology **12**, 1398 (2021). doi:10.3389/fphys.2021.716597
18. Alnæs, M.S., Blechta, J., Hake, J., Johansson, A., Kehlet, B., Logg, A., Richardson, C., Ring, J., Rognes, M.E., Wells, G.N.: The fenics project version 1.5. Archive of Numerical Software **3**(100) (2015). doi:10.11588/ans.2015.100.20553

19. Logg, A., Mardal, K.-A., Wells, G.N., *et al.*: Automated Solution of Differential Equations by the Finite Element Method. Springer, ??? (2012). doi:10.1007/978-3-642-23099-8
20. Karras, T., Laine, S., Aittala, M., Hellsten, J., Lehtinen, J., Aila, T.: Analyzing and Improving the Image Quality of StyleGAN. arXiv (2019). doi:10.48550/ARXIV.1912.04958
21. Karras, T., Aittala, M., Hellsten, J., Laine, S., Lehtinen, J., Aila, T.: Training Generative Adversarial Networks with Limited Data. arXiv (2020). doi:10.48550/ARXIV.2006.06676
22. Kingma, D.P., Ba, J.: Adam: A Method for Stochastic Optimization. arXiv (2014). doi:10.48550/ARXIV.1412.6980
23. Bloice, M., Roth, P., Holzinger, A.: Biomedical image augmentation using Augmentor. Bioinformatics **35**(21), 4522–4524 (2019). doi:10.1093/bioinformatics/btz259
24. Wissmann, L., Santelli, C., Segars, W.P., Kozerke, S.: Realistic numerical phantoms for cardiovascular magnetic resonance. Journal of Cardiovascular Magnetic Resonance **16**(1) (2014). doi:10.1186/s12968-014-0063-3
